# Early identification of neoadjuvant therapy non-response via multimodal immune-imaging biomarkers in breast cancer

**DOI:** 10.3389/fimmu.2026.1877547

**Published:** 2026-06-10

**Authors:** Xiangyuan Zhou, Xianming Huang, Lan Liu, Xiaoqin Cai, Han Li, Zhikang Sun, Zongqing Qiu, Jinxiu Zhong, Tenghua Yu, Qiao Zeng

**Affiliations:** 1 Department of Radiology, Jiangxi Cancer Hospital & Institute, Jiangxi Clinical Research Center for Cancer, The Second Affiliated Hospital of Nanchang Medical College, Nanchang, Jiangxi, China; 2 Department of Pathology, Jiangxi Cancer Hospital & Institute, Jiangxi Clinical Research Center for Cancer, The Second Affiliated Hospital of Nanchang Medical College, Nanchang, Jiangxi, China; 3 Nanchang Medical College, Nanchang, Jiangxi, China; 4 Department of Nuclear Medicine, Jiangxi Cancer Hospital & Institute, Jiangxi Clinical Research Center for Cancer, The Second Affiliated Hospital of Nanchang Medical College, Nanchang, Jiangxi, China; 5 Department of Breast Surgery, Jiangxi Cancer Hospital & Institute, Jiangxi Clinical Research Center for Cancer, The Second Affiliated Hospital of Nanchang Medical College, Nanchang, Jiangxi, China; 6 Department of Radiology, Jiangxi Cancer Hospital & Institute, Jiangxi Clinical Research Center for Cancer, The Second Affiliated Hospital of Nanchang Medical College, JXHC Key Laboratory of Tumour Metastasis (Jiangxi Cancer Hospital), Nanchang, Jiangxi, China

**Keywords:** breast cancer, magnetic resonance imaging, neoadjuvant therapy, non−response, pan−immune−inflammation value, tumor microenvironment, tumor−infiltrating lymphocytes

## Abstract

**Background:**

Early identification of breast cancer patients unlikely to benefit from neoadjuvant therapy (NAT) remains a critical unmet need. This study aimed to develop and internally validate a multimodal prediction model for NAT non−response by integrating clinicopathological, tumor microenvironment (TME), longitudinal magnetic resonance imaging (MRI), and systemic inflammatory features.

**Methods:**

In this retrospective study, 112 patients with primary breast cancer underwent baseline MRI, a second MRI after two NAT cycles, and definitive surgery. Non−response was defined as Miller–Payne grades 1–3. Candidate predictors were categorized into four domains. After univariate screening, domain−specific multivariable logistic regression was performed, and retained variables entered least absolute shrinkage and selection operator (LASSO) regression to construct a final multimodal model. Internal validation included five-fold cross−validation and 500−iteration bootstrap. Calibration and decision curve analyses were also performed.

**Results:**

Thirty−eight patients (33.9%) were non−responders. The individual domain models achieved apparent AUCs of 0.844 (clinical), 0.786 (imaging), 0.828 (TME), and 0.706 (inflammatory). Following LASSO selection, nine features were retained: HER2 status, ER status, Ki−67 index, late enhancement rate after two cycles (LER2), baseline background parenchymal enhancement (BPE), time to peak after two cycles (TTP2), tumor−stroma ratio (TSR), tumor−infiltrating lymphocytes (TILs), and pan−immune−inflammation value after two cycles (PIV2). The multimodal model yielded an apparent AUC of 0.933 (95% CI: 0.890–0.977), with a bootstrap−corrected AUC of 0.855 and a mean five-fold cross−validation AUC of 0.908 ± 0.038. TILs, TSR, PIV2, and Ki−67 were independent predictors. The model demonstrated acceptable calibration after correction for optimism and a net clinical benefit across a range of thresholds.

**Conclusions:**

A multimodal prediction model integrating clinicopathological, imaging, tumor microenvironment, and systemic inflammatory features showed potential for early identification of breast cancer patients unlikely to benefit from neoadjuvant therapy. However, given the limited sample size and exploratory single-center design, performance estimates should be interpreted cautiously, and external validation is essential.

## Introduction

For patients with locally advanced or high-risk early-stage breast cancer, neoadjuvant therapy (NAT) has become an established component of multimodal treatment (1). In addition to reducing tumor burden and increasing the feasibility of breast-conserving surgery, NAT provides *in vivo* information on treatment sensitivity through pathological response assessment. However, a clinically meaningful subset of patients derives limited benefit from NAT and fails to achieve substantial tumor regression (2). Continued exposure to ineffective treatment may delay timely treatment adaptation and increase unnecessary toxicity (3). Therefore, early identification of patients at high risk of NAT non-response remains an important unmet need in precision breast cancer management.

Multiparametric magnetic resonance imaging (mpMRI), integrating T2-weighted imaging, diffusion-weighted imaging (DWI), and dynamic contrast-enhanced MRI (DCE-MRI), provides complementary information on tumor morphology, diffusion restriction, and vascular perfusion (4). mpMRI has been widely used to monitor treatment response in breast cancer, and both baseline and on-treatment MRI features, including enhancement kinetics and apparent diffusion coefficient (ADC), have shown value for predicting pathological response (5). Nevertheless, most previous studies have focused on single imaging parameters or single time points, and the utility of early longitudinal MRI changes for identifying patients who are unlikely to benefit from NAT remains insufficiently defined.

In addition, increasing evidence indicates that treatment sensitivity is influenced not only by tumor−intrinsic characteristics but also by systemic inflammatory status and the tumor microenvironment (TME). Peripheral blood inflammatory indices, such as neutrophil-to-lymphocyte ratio (NLR), monocyte-to-lymphocyte ratio (MLR), platelet-to-lymphocyte ratio (PLR), systemic immune-inflammation index (SII), systemic inflammation response index (SIRI), and pan-immune-inflammation value (PIV), are accessible biomarkers reflecting host-tumor interactions and have been associated with NAT response and prognosis in breast cancer (6). Longitudinal assessments of these indices, whether as absolute on−treatment values or as dynamic changes, may reflect early host responses to therapy and have been associated with treatment outcomes (7). Tumor-infiltrating lymphocytes (TILs) and tumor-stroma ratio (TSR) reflect critical immune and stromal characteristics of the TME and have been linked to therapeutic response and clinical outcomes (8, 9).

Despite these advances, most existing studies have evaluated imaging, inflammatory biomarkers, or TME-related factors, and many have used pathological complete response as the primary endpoint. In clinical practice, however, early identification of patients who are unlikely to achieve meaningful tumor regression may be equally important, because it may help guide closer monitoring and earlier treatment adjustment. Studies specifically addressing non−response, defined by Miller–Payne grade 1–3, and integrating early longitudinal mpMRI features, systemic inflammatory status, and TME immune characteristics remain scarce. A framework that jointly captures tumor−intrinsic biology, local immune contexture, and treatment−induced systemic immune perturbations may offer a more holistic approach to identify resistant cases early. Therefore, this study retrospectively collected multimodal data to develop and internally validate an early prediction model for NAT non-response in breast cancer.

## Materials and methods

### Study design and patients

**Figure 1:**
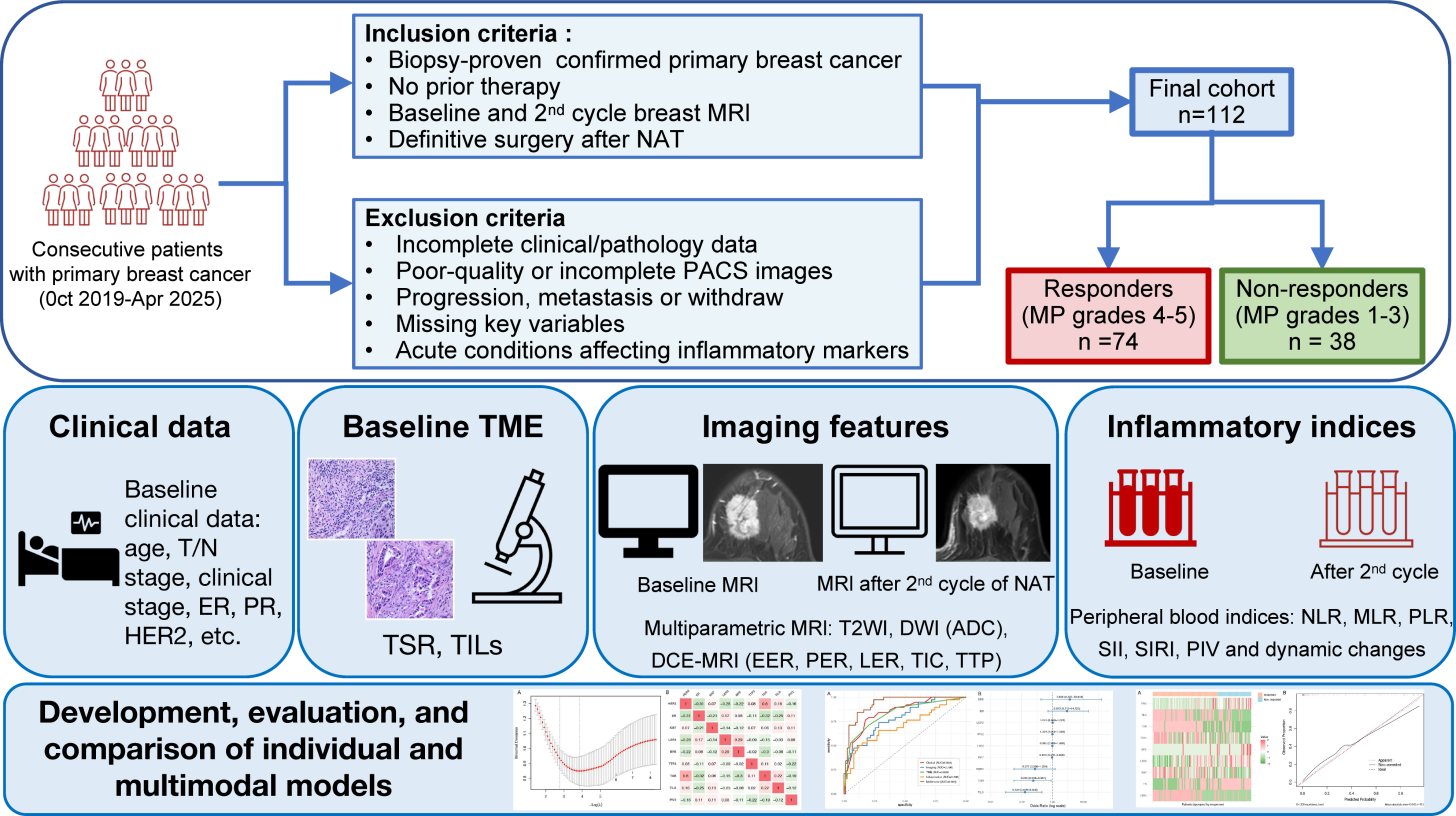
Study workflow and patient selection. Flow diagram illustrating the patient enrollment process. Patients with primary breast cancer who received NAT between October 2019 and April 2025 were consecutively screened according to the inclusion and exclusion criteria. Eligible patients completed baseline and two-cycle breast MRI, peripheral blood sampling, and definitive surgery. Candidate predictors were grouped into four domains (clinicopathological, imaging, tumor microenvironment, and inflammatory). Individual models were built within each domain, and retained variables were pooled for LASSO regression to select features for the final multimodal model. The multimodal model was internally validated and compared with each individual model. NAT, neoadjuvant therapy; MRI, magnetic resonance imaging; PACS, picture archiving and communication system; LASSO, least absolute shrinkage and selection operator; AUC, area under the receiver operating characteristic curve; TILs, tumor-infiltrating lymphocytes; TSR, tumor-stroma ratio.

The study workflow and patient selection process are summarized in **Figure 1**. This retrospective cohort study consecutively enrolled patients with primary breast cancer who received NAT at our institution between October 2019 and April 2025. Inclusion criteria were: (1) pathologically confirmed primary breast cancer on pretreatment core needle biopsy, without prior anticancer therapy; (2) breast MRI and peripheral blood sampling performed both at baseline and after two cycles of NAT; and (3) definitive surgery completed after NAT. Exclusion criteria were: (1) incomplete clinical or pathological data; (2) incomplete or poor-quality PACS images precluding imaging assessment; (3) disease progression, metastasis, or premature withdrawal during treatment; (4) missing key variables that could not be reliably retrieved; and (5) non-tumor-related conditions within 2 weeks before blood sampling that might affect inflammatory indices.

### Clinical data and pathological assessment

Baseline clinical data were extracted from electronic medical records, including age, tumor location, clinical T stage, clinical N stage, overall clinical stage, serum tumor markers.

Baseline core needle biopsy specimens and postoperative surgical specimens were fixed in formalin, embedded in paraffin, and stained with hematoxylin and eosin, with additional immunohistochemistry performed when indicated. HER2 positivity was defined as IHC 3+ or IHC 2+ with fluorescence *in situ* hybridization−confirmed gene amplification. ER and PR positivity were defined as nuclear staining in at least 1% of tumor cells.

Given the substantial heterogeneity of neoadjuvant chemotherapy regimens, treatment protocols were categorized into four major groups according to chemotherapy backbone and HER2-targeted therapy status: anthracycline-taxane sequential regimens, HER2-targeted regimens, platinum/taxane-based regimens, and other regimens. Detailed original regimens are summarized in **Supplementary Table 1**. Treatment category was summarized descriptively and compared between groups but was not incorporated into the final multivariable model because of strong biological correlation with molecular subtype and the limited sample size within individual regimens.

TILs were assessed according to the 2014 International TILs Working Group recommendations. The average percentage of stromal TILs was recorded, and TILs were categorized as low (<10%) or high (≥10%) (10). TSR was categorized as stroma−rich (≥50%) or stroma−poor (<50%) (11, 12). Miller–Payne grading was used to evaluate NAT response: grades 4–5 were defined as responders, and grades 1–3 as non−responders. All pathological slides were reviewed by a senior breast pathologist blinded to clinical and imaging data according to standardized institutional pathological assessment criteria.

### MRI acquisition and imaging assessment

All patients underwent breast MRI at baseline and after two cycles of NAT using a 3.0−T scanner (Philips Ingenia 3.0T) with a 16−channel dedicated bilateral breast coil. The protocol included T2−weighted imaging with fat suppression, DWI (b = 0 and 1000 s/mm²), and DCE−MRI. ADC maps were generated automatically. Time−intensity curve (TIC) analysis and maximum intensity projection images were derived from post−processing. For multifocal disease, the largest lesion was selected. The following MRI features were recorded at both time points: maximum tumor diameter, background parenchymal enhancement (BPE), lesion type, peritumoral edema, intratumoral necrosis, ADC, TIC pattern, early enhancement rate (EER), peak enhancement rate (PER), late enhancement rate (LER), and time to peak (TTP). Two experienced breast radiologists (>10 years of experience), blinded to pathological outcomes, independently reviewed all MRI images. In cases of discrepant interpretation, consensus was reached through joint review.

### Reproducibility assessment

To evaluate the reproducibility of MRI interpretation, the two experienced breast radiologists independently re-evaluated all baseline and post-2-cycle images while blinded to pathological outcomes and each other’s readings. Inter-observer agreement for continuous variables was assessed using the intraclass correlation coefficient (ICC; two-way random-effects, absolute agreement, single rater). For categorical variables, Cohen’s kappa was used for binary features and weighted kappa (quadratic weights) for ordered categorical features (TIC type). For pathological assessment, TILs and TSR were evaluated by a single senior breast pathologist. To assess intra-observer reproducibility, the same pathologist re-evaluated all pretreatment biopsy slides after the 8-week washout period, blinded to the original readings and clinical data. Agreement for these binary classifications was assessed using Cohen’s kappa. Interpretation of agreement strength followed the Landis and Koch criteria.

### Peripheral blood inflammatory indices

Peripheral blood counts obtained at baseline and after two cycles of NAT were collected. Derived inflammatory indices included NLR, MLR, PLR, SII, SIRI, and PIV. Dynamic changes for each index were defined as the post-2-cycle value minus the baseline value. These changes are denoted with the prefix “d” (e.g., dNLR, dMLR, dPLR, dSII, dSIRI, dPIV).

### Statistical analysis

Continuous variables are presented as mean ± standard deviation or median (interquartile range), and categorical variables as frequencies (percentages). Between-group comparisons used the t-test, Mann–Whitney U test, χ² test, or Fisher’s exact test as appropriate.

Feature selection was performed in two steps. First, candidate variables were grouped into four domains: (1) clinicopathological (age, tumor location, T, N, stage, CA15-3, CA125, ER, PR, HER2, Ki-67); (2) imaging (baseline and post-2-cycle MRI features); (3) tumor microenvironment (TILs, TSR); and (4) inflammatory (baseline, post-2-cycle, and dynamic indices). Within each domain, variables with *P* < 0.05 in univariate logistic regression were entered into a multivariable stepwise logistic regression to build an individual model. Retained variables were pooled and subjected to least absolute shrinkage and selection operator (LASSO) regression to select features for the final multimodal logistic model. All variable selection steps were performed on the entire dataset prior to cross-validation; the selection procedure was not nested within the cross-validation folds. Variables with non-zero coefficients were used to construct the final multimodal logistic regression model. Bootstrap-based variable stability analysis was additionally performed to evaluate the consistency of feature selection across resampling iterations. Feature selection and model construction were performed on the overall training cohort, followed by internal five-fold cross-validation for performance estimation. We acknowledge that feature selection was not fully repeated within each cross-validation fold, which may introduce a degree of optimism bias. Model performance was evaluated by the area under the receiver operating characteristic curve (AUC) with 95% confidence intervals. AUCs of the individual models and the multimodal model were compared using DeLong’s test. All tests were two-sided, and *P* < 0.05 was considered statistically significant. To further evaluate model stability and potential overfitting, bootstrap-based internal validation and calibration analysis were additionally performed. Model calibration was assessed using calibration plots, Brier score, calibration intercept, and calibration slope. Decision curve analysis (DCA) was conducted to evaluate the potential clinical utility of the multimodal model. Multicollinearity among retained variables was assessed using variance inflation factors (VIF). Analyses were performed with R version 4.3.1 and Python 3.9.5.

## Results

### Baseline characteristics and development of individual models

A total of 112 patients were included (74 responders, 38 non−responders). Non-responders had lower rates of ER negativity, PR negativity, and HER2 positivity, and a lower Ki-67 index (all P < 0.05). Responders had significantly higher proportions of high TILs (≥10%) and stroma-poor status than non-responders (both P < 0.001) (**Table 1**). Significant differences in neoadjuvant treatment category distribution were observed between the response and non-response groups (P < 0.001). HER2-targeted regimens were more frequently administered in HER2-positive tumors, whereas platinum/taxane-based regimens were more commonly used in triple-negative breast cancer.

**Table 1:** Baseline clinicopathological characteristics between responders and non-responders.

Characteristic	Non-responders (n=38)	Responders (n=74)	P value
Age (years)	47.45 ± 10.78	51.00 ± 8.73	0.063
Tumor location [n (%)]			0.587
Left	19 (50.0)	41 (55.4)	
Right	19 (50.0)	33(44.6)	
T stage [n (%)]			0.508
T1	5 (13.2)	7 (9.5)	
T2	23 (60.5)	54 (73.0)	
T3	8 (21.1)	9 (12.2)	
T4	2 (5.3)	4 (5.4)	
N stage [n (%)]			0.834
N0	7 (18.4)	10 (13.5)	
N1	12 (31.6)	29 (39.2)	
N2	14 (36.8)	25 (33.8)	
N3	5 (13.2)	10 (13.5)	
Clinical stage [n (%)]			0.076
I	2 (5.3)	1 (1.4)	
II	13 (34.2)	32 (43.2)	
III	19 (50.0)	40 (54.1)	
IV	4 (10.5)	1 (1.4)	
CA15-3 (U/mL)	9.88 (8.00, 19.85)	9.95 (7.00, 19.30)	0.919
CA125 (U/mL)	13.07 (9.53, 17.52)	13.41 (10.46, 18.45)	0.988
ER status [n (%)]			<0.001
Positive	33 (86.8)	35 (47.3)	
Negative	5 (13.2)	39 (52.7)	
PR status [n (%)]			<0.001
Positive	31 (81.6)	31 (41.9)	
Negative	7 (18.4)	43 (58.1)	
HER2 status [n (%)]			<0.001
Positive	7 (18.4)	50 (67.6)	
Negative	31 (81.6)	24 (32.4)	
Ki-67 index (%)	30.0 (20.0, 57.5)	55.0 (30.0, 70.0)	0.005
TILs level [n (%)]			<0.001
Low (<10%)	27 (71.1)	21 (28.4)	
High (≥10%)	11 (28.9)	53 (71.6)	
TSR [n (%)]			<0.001
Stroma-rich (≥50%)	27 (71.1)	15 (20.3)	
Stroma-poor (<50%)	11 (28.9)	59 (79.7)	
Treatment category [n (%)]			<.001
Anthracycline-taxane sequential	28 (73.68)	15 (20.27)	
HER2-targeted	6 (15.79)	36(48.56)	
Platinum-based TP	3 (7.89)	21 (28.38)	
Other	1 (2.63)	2 (2.70)	

Data are presented as mean ± SD for normally distributed continuous variables, median (IQR) for non normally distributed continuous variables, or frequency (percentage) for categorical variables. Non-responders: Miller–Payne grades 1–3; responders: Miller–Payne grades 4–5. ER, estrogen receptor; PR, progesterone receptor; HER2, human epidermal growth factor receptor 2; TILs, tumor infiltrating lymphocytes; TSR, tumor stroma ratio.

Baseline peripheral blood inflammatory indices did not differ significantly between groups (**Table 2**). After two cycles of NAT, several imaging and inflammatory variables showed significant differences (**Tables 2**, **3**). Among inflammatory indices, post−treatment neutrophil count, monocyte count, platelet count, NLR, MLR, SII, SIRI, PIV, and dynamic changes dNLR, dMLR, dPLR, dSII, dSIRI, dPIV were all significantly elevated in non−responders (**Table 2**). Responders had lower post−treatment EER, PER, LER, higher ADC, and longer TTP. TIC pattern distribution also differed (all P < 0.05). Detailed imaging comparisons are provided in **Table 3**.

**Table 2:** Comparison of peripheral blood inflammatory indices between responders and non-responders.

Variable	Non-responders (n=38)	Responders (n=74)	P value
Baseline blood cell counts			
Neutrophil count (×10^9^/L)	3.48 (2.90, 4.63)	3.50 (2.77, 4.66)	0.601
Lymphocyte count (×10^9^/L)	1.79 (1.37, 1.98)	1.48 (1.29, 1.78)	0.136
Monocyte count (×10^9^/L)	0.31 (0.23, 0.39)	0.31 (0.25, 0.38)	0.949
Platelet count (×10^9^/L)	244.50 (200.75, 301.25)	226.50 (193.00, 277.75)	0.390
Blood cell counts after two cycles			
Neutrophil count (×10^9^/L)	4.04 (3.00, 5.40)	2.71 (1.85, 4.19)	0.005
Lymphocyte count (×10^9^/L)	1.27 (0.94, 1.68)	1.28 (1.05, 1.57)	0.848
Monocyte count (×10^9^/L)	0.47 (0.33, 0.65)	0.35 (0.24, 0.44)	0.001
Platelet count (×10^9^/L)	299.00 (221.50, 370.00)	243.50 (196.75, 311.25)	0.037
Baseline inflammatory indices			
NLR	2.19 (1.87, 2.94)	2.20 (1.81, 3.05)	0.993
MLR	0.19 (0.15, 0.22)	0.20 (0.16, 0.26)	0.286
PLR	145.72 (118.32, 172.20)	143.54 (118.78, 186.36)	0.885
SII	511.97 (360.16, 715.99)	483.52 (342.74, 753.68)	0.634
SIRI	0.65 (0.49, 0.89)	0.72 (0.44, 1.04)	0.939
PIV	134.64 (92.46, 274.45)	138.65 (92.56, 269.13)	0.813
Inflammatory indices after two cycles			
NLR	3.15 (2.09, 4.42)	2.08 (1.46, 3.14)	0.008
MLR	0.38 (0.27, 0.52)	0.25 (0.18, 0.37)	0.002
PLR	234.92 (168.52, 340.80)	199.03 (144.37, 280.80)	0.070
SII	1050.29 (439.37, 1405.89)	533.38 (325.64, 908.47)	0.004
SIRI	1.72 (0.98, 2.52)	0.69 (0.37, 1.23)	<0.001
PIV	408.51 (261.19, 888.79)	165.14 (80.57, 319.46)	<0.001
Dynamic changes			
dNLR	0.70 (–0.37, 2.17)	–0.11 (–0.95, 0.86)	0.010
dMLR	–0.25 (–0.70, 0.20)	0.20 (–0.20, 0.72)	0.003
dPLR	81.89 (8.45, 179.27)	43.46 (–2.46, 109.24)	0.027
dSII	388.47 (–88.91, 928.59)	60.97 (–184.48, 358.48)	0.005
dSIRI	0.85 (0.16, 1.75)	–0.02 (–0.38, 0.45)	<0.001
dPIV	282.20 (39.99, 673.29)	22.66 (–68.16, 170.86)	<0.001

NLR, neutrophil-to-lymphocyte ratio; MLR, monocyte-to-lymphocyte ratio; PLR, platelet-to-lymphocyte ratio; SII, systemic immune-inflammation index (platelet × neutrophil/lymphocyte); SIRI, systemic inflammation response index (neutrophil × monocyte/lymphocyte); PIV, pan-immune-inflammation value (neutrophil × monocyte × platelet/lymphocyte); d, dynamic change (post-2nd minus baseline).

**Table 3:** Comparison of imaging features at baseline and after two cycles of NAT between responders and non-responders.

Variable	Non-responders (n=38)	Responders (n=74)	P value
Baseline MRI features			
D1 (cm)	3.10 (2.50, 4.00)	3.05 (2.52, 3.88)	0.618
D2 (cm)	2.00 (1.42, 3.48)	1.60 (1.00, 2.27)	0.011
Dd (%)	35.50 (18.65, 51.90)	53.45 (29.65, 71.05)	0.003
BPE [n (%)]			0.007
Minimal/mild	30 (78.9)	71 (95.9)	
Moderate/marked	8 (21.1)	3 (4.1)	
Lesion type [n (%)]			0.918
Mass	24 (63.2)	46 (62.2)	
Non-mass	14 (36.8)	28 (37.8)	
Edema present [n (%)]	31 (81.6)	66 (89.2)	0.263
Necrosis present [n (%)]	17 (44.7)	36 (48.6)	0.695
TIC type [n (%)]			0.152
Type I (persistent)	0 (0.0)	1 (1.4)	
Type II (plateau)	4 (10.5)	17 (23.0)	
Type III (washout)	34 (89.5)	56 (75.7)	
EER (%)	105.50 (63.25, 154.75)	116.50 (88.75, 145.75)	0.519
PER (%)	204.00 (156.25, 242.50)	184.00 (142.00, 237.50)	0.241
LER (%)	157.50 (124.25, 190.75)	149.00 (125.50, 195.75)	1.000
TTP (s)	160.00 (147.25, 230.00)	170.00 (145.00, 234.50)	0.995
ADC (×10⁻³ mm²/s)	0.73 (0.64, 0.85)	0.81 (0.71, 0.92)	0.008
MRI features after two cycles			
Post-2nd BPE change [n (%)]			0.608
No obvious change	30 (78.9)	64 (86.5)	
Change	8 (21.1)	10 (13.5)	
Post-2nd Edema present [n (%)]	20 (52.6)	21 (28.4)	0.012
Post-2nd Necrosis present [n (%)]	10 (26.3)	11 (14.9)	0.142
Post-2nd TIC type [n (%)]			<0.001
Type I (persistent)	0 (0.0)	26 (35.1)	
Type II (plateau)	14 (36.8)	22 (29.7)	
Type III (washout)	24 (63.2)	26 (35.1)	
Post-2nd EER (%)	82.50 (55.25, 134.75)	37.50 (14.25, 98.75)	0.001
Post-2nd PER (%)	175.00 (131.50, 213.75)	136.50 (92.00, 183.50)	0.003
Post-2nd LER (%)	152.50 (121.25, 192.50)	119.00 (74.25, 165.00)	0.004
Post-2nd TTP (s)	220.50 (162.50, 380.75)	365.50 (210.75, 450.00)	0.013
Post-2nd ADC (×10⁻³ mm²/s)	0.94 (0.79, 1.04)	1.06 (0.86, 1.35)	0.022

D1, maximum tumor diameter at baseline; D2, maximum tumor diameter after two cycles; Dd, tumor regression ratio; BPE, background parenchymal enhancement; TIC, time-intensity curve; EER, early enhancement rate; PER, peak enhancement rate; LER, late enhancement rate; TTP, time to peak; ADC, apparent diffusion coefficient.

Inter-observer agreement for MRI variables ranged from substantial to almost perfect (ICC 0.70–0.84; kappa 0.74–0.86; weighted kappa 0.79–0.80). The highest agreements were observed for lesion type (κ = 0.86) and ADC2 (ICC = 0.84). Intra-observer reproducibility for pathological TILs and TSR was almost perfect (κ = 0.87 and 0.83, respectively). Detailed agreement metrics for all evaluated variables are provided in **Supplementary Table 2**.

In the stepwise regression within each domain, the clinicopathological model retained HER2, ER, and Ki−67 (AUC = 0.844, 95% CI: 0.768–0.920). The imaging model retained LER2, baseline BPE, baseline ADC, and TTP2 (AUC = 0.786, 95% CI: 0.697–0.874). The TME model retained TSR and TILs (AUC = 0.828, 95% CI: 0.748–0.909). The inflammatory model retained PIV2, dSIRI, and SIRI2 (AUC = 0.706, 95% CI: 0.598–0.813). All individual models are summarized in **Table 4**.

**Table 4:** Diagnostic performance of all models.

Model	AUC	95% CI	Sensitivity	Specificity	PPV	NPV	Accuracy	P value
Clinical	0.844	0.768 – 0.920	0.763	0.824	0.690	0.871	0.804	<0.01
Imaging	0.786	0.697 – 0.874	0.737	0.716	0.571	0.841	0.723	<0.001
TME	0.828	0.748 – 0.909	0.711	0.797	0.643	0.843	0.768	<0.01
Inflammation	0.706	0.598 – 0.813	0.500	0.878	0.679	0.774	0.750	<0.001
Multimodal	0.933	0.890 – 0.977	0.842	0.892	0.800	0.917	0.875	NA

AUC, area under the receiver operating characteristic curve; CI, confidence interval; PPV, positive predictive value; NPV, negative predictive value. All performance metrics are apparent estimates derived from the full training dataset. For the multimodal model, internal five-fold cross-validation yielded a mean AUC of 0.908 ± 0.038, and 500-iteration bootstrap validation gave an optimism-corrected AUC of 0.855. These corrected estimates indicate a non-negligible degree of optimism in the apparent AUC of 0.933. P value refers to comparisons between each individual model and the Multimodal model via DeLong test.

### Feature selection and development of the multimodal model

**Figure 2:**
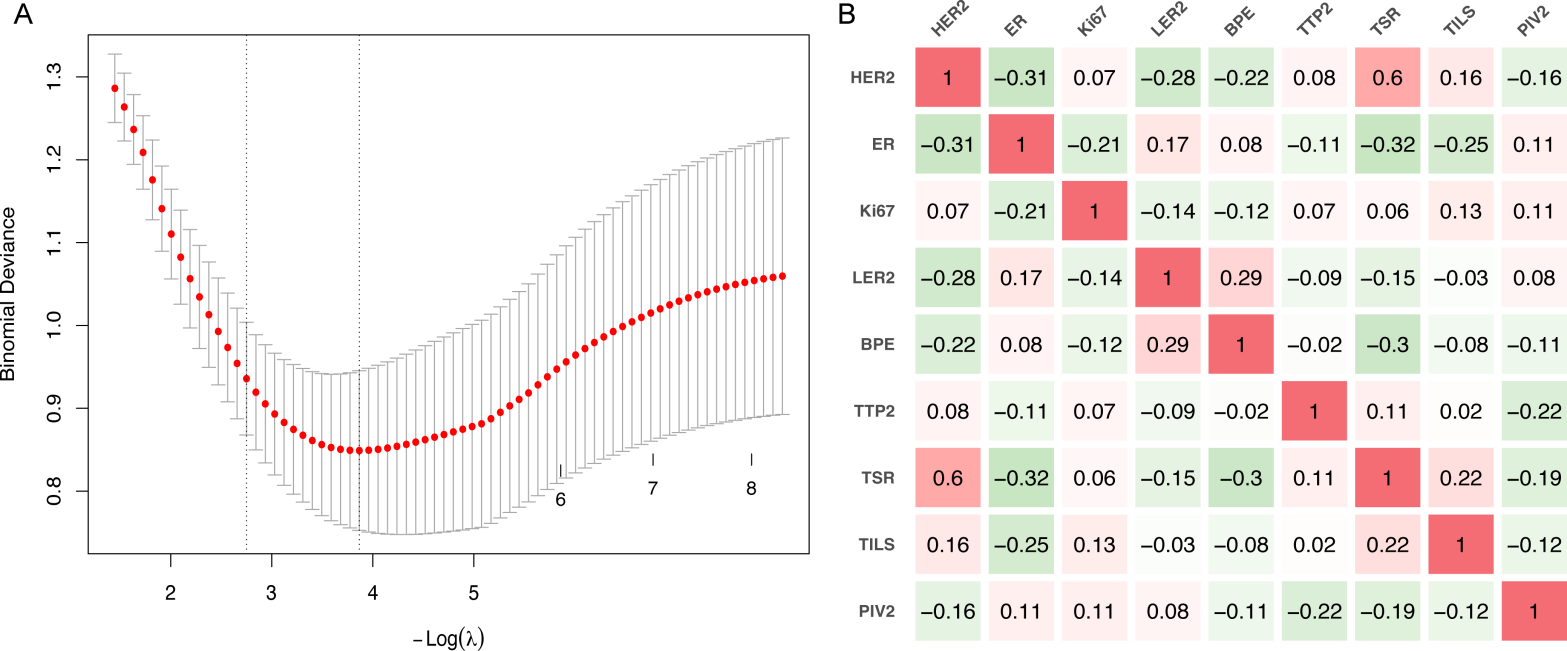
Feature selection using LASSO regression and correlation analysis of the selected variables. **(A)** LASSO coefficient profiles of the 12 candidate variables retained by the four individual models. The optimal penalty parameter (λ) was determined by 10-fold cross-validation (lambda.min). Nine variables with non-zero coefficients were selected for the final multimodal model. **(B)** Spearman correlation heatmap of the nine selected variables. All pairwise correlation coefficients were low (|ρ| < 0.75). LASSO, least absolute shrinkage and selection operator; HER2, human epidermal growth factor receptor 2; ER, estrogen receptor; LER2, late enhancement rate after two cycles; BPE, background parenchymal enhancement; TTP2, time to peak after two cycles; TSR, tumor-stroma ratio; TILs, tumor-infiltrating lymphocytes; PIV2, pan-immune-inflammation value after two cycles.

**Figure 3:**
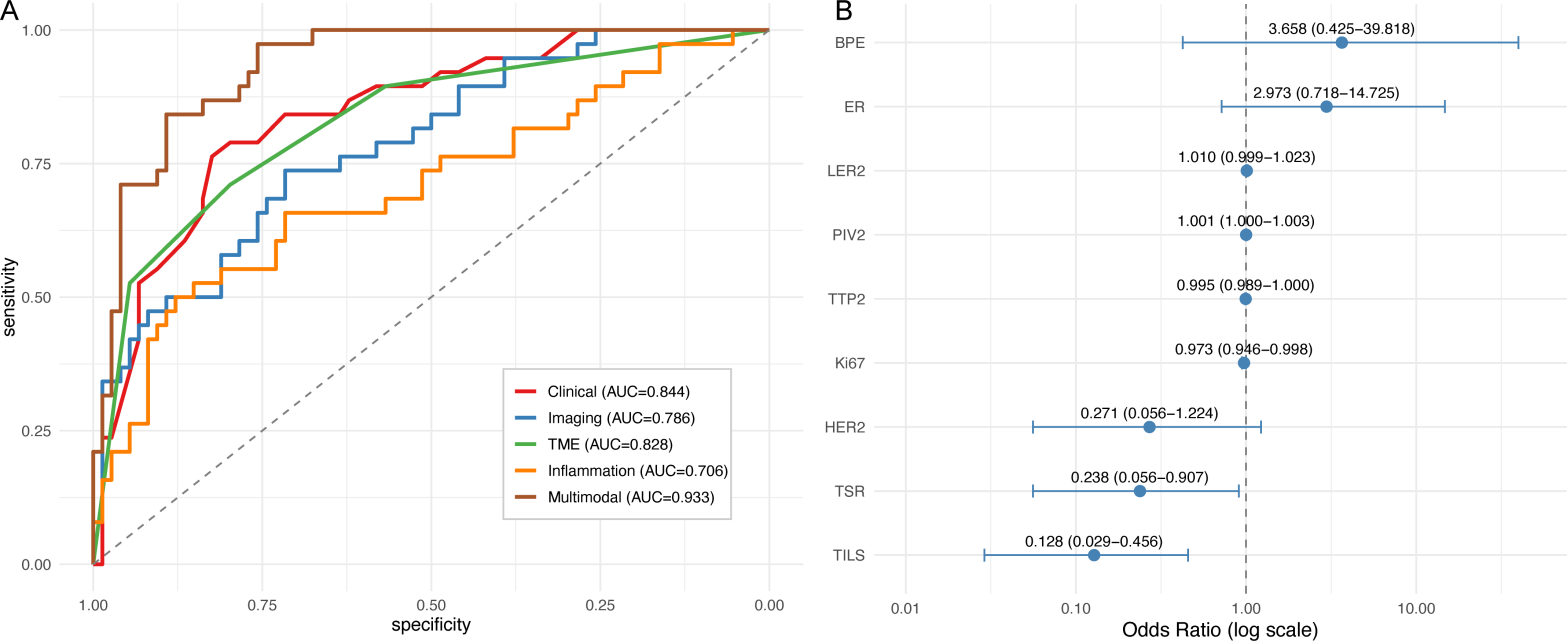
Models comparison and multivariable odds ratios of the multimodal model. **(A)** Receiver operating characteristic (ROC) curves comparing the multimodal model with the four individual domain models (clinical, imaging, TME, inflammatory). **(B)** Forest plot showing the odds ratios and 95% confidence intervals for all nine variables included in the final multimodal model. Statistically significant independent predictors (P < 0.05) are indicated by confidence intervals not crossing 1.0 and are noted in the main text (TILs, TSR, Ki-67, PIV2). HER2, human epidermal growth factor receptor 2; ER, estrogen receptor; LER2, late enhancement rate after two cycles; BPE, background parenchymal enhancement; TTP2, time to peak after two cycles; TSR, tumor-stroma ratio; TILs, tumor-infiltrating lymphocytes; PIV2, pan-immune-inflammation value after two cycles.

**Figure 4:**
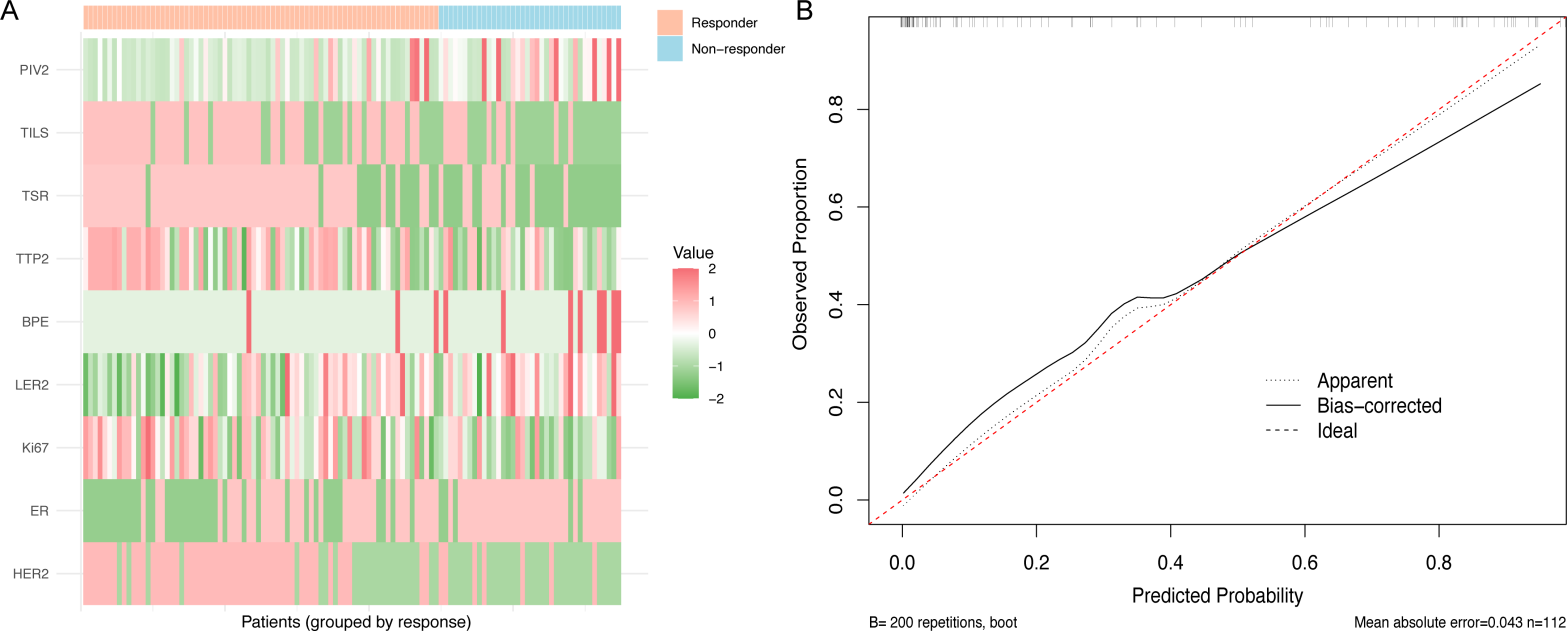
Heatmap and calibration of the multimodal model. **(A)** Heatmap of the nine selected variables (rows) across 112 patients (columns). Patients are grouped by true response status (Responder vs. Non-responder, top annotation bar) and within each group sorted by increasing predicted probability of non-response. Colors represent z-scored variable values (green: low; pink: high). **(B)** Bootstrap optimism-corrected calibration curve (200 resamples). The dashed diagonal line represents perfect calibration; the solid line shows the bias-corrected relationship between predicted probability and observed proportion. Mean absolute error = 0.043.

The twelve variables retained by the four individual models were subjected to LASSO regression (**Figure 2A**). Nine variables with non-zero coefficients were selected: HER2, ER, Ki-67, LER2, BPE, TTP2, TSR, TILs, and PIV2. Pairwise correlations among these features were low (**Figure 2B**). These nine variables were entered into the multimodal model. On the full training set, the model showed excellent apparent discrimination with an AUC of 0.933 (95% CI: 0.890–0.977), which was significantly higher than each individual model (P < 0.01, DeLong’s test) (**Table 4**; **Figure 3A**). To obtain a more realistic estimate of performance, internal validation was performed using five-fold cross-validation (without re-selection of features) and 500-iteration bootstrap. Five-fold cross-validation yielded a mean AUC of 0.908 ± 0.038, and bootstrap internal validation gave a corrected AUC of 0.855, indicating a non-negligible degree of optimism in the apparent estimate. In multivariable analysis, TILs (OR = 0.128, P = 0.003), TSR (OR = 0.238, P = 0.040), PIV2 (OR = 1.001, P = 0.021), and Ki-67 (OR = 0.973, P = 0.044) were independently associated with non-response (**Figure 3B**). The patient-level heatmap demonstrated a clear separation of feature patterns between responders and non-responders (**Figure 4A**).

Model calibration was assessed in two complementary ways. The apparent calibration metrics on the full dataset were with Brier score = 0.099, calibration intercept = 0, calibration slope = 1. These perfect values likely reflect overfitting given the limited sample size. In contrast, bootstrap-based calibration (200 resamples) yielded a mean absolute error of 0.043 and a calibration curve (**Figure 4B**) that showed reasonable agreement between predicted and observed probabilities after correction for optimism. No significant multicollinearity was observed among retained variables (all VIF < 1.5; **Supplementary Table 3**). Bootstrap-based variable stability analysis demonstrated that core features including TILs, HER2, Ki-67, TSR, and TTP2 were consistently selected across resamples (**Supplementary Table 4**), supporting the relative stability of the predictive signature. Decision curve analysis further demonstrated that the multimodal model provided favorable net clinical benefit across a broad range of threshold probabilities (**Supplementary Figure 1**).

## Discussion

In this retrospective study, we developed and internally validated a multimodal model integrating longitudinal MRI features, systemic inflammatory indices, and tumor microenvironment characteristics for early prediction of NAT non-response in breast cancer. The final multimodal model achieved an apparent AUC of 0.933, although bootstrap internal validation indicated a non-negligible degree of optimism (corrected AUC = 0.855). These findings suggest that therapeutic resistance is driven not only by tumor-intrinsic biology, but also by dynamic interactions among vascular remodeling, systemic inflammation, and the immune microenvironment during treatment.

The final multimodal model for predicting non-response retained two semiquantitative DCE-MRI kinetic parameters (LER2 and TTP2) and baseline BPE. In this model, after two treatment cycles, non-responders had persistently short TTP2 and elevated LER2, whereas responders showed significantly longer TTP2 and lower LER2. This contrast in kinetic behavior likely reflects differences in vascular remodeling. In non-responders, short TTP2 suggests failure of therapy-induced vascular normalization and ongoing perfusion heterogeneity (13), while elevated LER2 indicates sustained vascular permeability and immature neovasculature (14). In responders, prolonged TTP2 is consistent with effective treatment: tumor cell death and vascular collapse delay contrast arrival and peak enhancement (15). These interpretations are supported by recent evidence showing that TTP prolongation during neoadjuvant chemotherapy correlates with pathologic complete response (13), and that dynamic vascular features predict pathological response and recurrence patterns (validation AUC 0.892) (14). In addition, abnormal tumor vasculature has increasingly been linked to immune suppression and impaired drug delivery (16, 17), further supporting the biological significance of these imaging observations. Regarding BPE, which reflects hormonally mediated vascular activity in normal breast tissue, baseline BPE was retained in the final multimodal model as one of the selected predictive features. A 2025 systematic review of 13 eligible studies confirmed growing interest in BPE as a predictive biomarker for NAC response and highlighted a shift toward longitudinal assessment (18). Another large cohort study reported that higher diagnostic BPE independently predicted worse invasive disease-free survival (19, 20).

Consistent with previous studies, TILs remained one of the most consistently identified protective predictors against non-response, underscoring the importance of baseline immune microenvironment for effective therapy. A 2025 multicenter Chinese population study (n = 424) identified 10% as the optimal TILs threshold for predicting pCR, with high TILs associated with significantly higher pCR rates (29.3% vs. 8.7%; P < 0.001) (10). Real world data further confirm that high stromal TILs are strongly associated with pCR to neoadjuvant therapy (56.0% vs. 20.0%), particularly in HER2 positive and triple negative subtypes (21). A recent phase II study further demonstrated that pre-neoadjuvant treatment with decitabine plus pembrolizumab significantly increased stromal TIL levels and PD-L1 expression in HER2-negative breast cancer, suggesting that epigenetic modulation combined with immune checkpoint blockade may enhance antitumor immune activation and improve sensitivity to subsequent NAT (22). In contrast, TSR independently contributed to non-response prediction, suggesting that stromal composition represents another critical determinant of therapeutic sensitivity. Stroma-rich tumors are frequently associated with extracellular matrix remodeling, hypoxia, impaired infiltration of immune cells, and immunosuppression mediated by fibroblasts. TSR is seen to be independently associated with neoadjuvant chemotherapy resistance across breast cancer subtypes (23). Stroma-low patients significantly more often achieved (near) pCR of the tumor and axilla after NAT compared to stroma-high patients (24). Both TILs and TSR were assessed on baseline core needle biopsies, which sample only a localized tumor region. TSR is particularly vulnerable to sampling heterogeneity. Nevertheless, biopsy-based TIL evaluation has been well validated, showing consistent predictive value and excellent agreement with surgical specimens (ICC 0.895) (25). For TSR, a moderate biopsy–resection concordance has been reported (κ = 0.514) (11), yet biopsy TSR still provides meaningful stromal assessment (26). In this study, an experienced breast pathologist selected the most representative tumor area per standardized criteria, thereby minimizing, though not eliminating, sampling bias. Importantly, TILs and TSR likely reflect complementary biological processes. The coexistence of low TILs and abundant stromal architecture may represent a phenotype characterized by immune exclusion, which is associated with poor treatment sensitivity. A combined analysis of TILs and TSR confirmed that this approach using both markers is useful for determining prognosis and guiding treatment decisions in breast cancer (27).

A particularly important finding of this study was the independent predictive value of PIV after two cycles of NAT. Unlike baseline inflammatory markers, the inflammatory state at this early time point may better reflect real−time host–tumor interactions during the initial phase of therapy. PIV integrates neutrophil, monocyte, platelet, and lymphocyte counts into a composite marker reflecting the balance between pro−tumor inflammation and antitumor immunity. Persistent elevation of PIV after two cycles may indicate ongoing myeloid−driven inflammation and ineffective immune reprogramming early in the treatment course. A multicenter study (n = 507) confirmed that PIV is an independent predictor of pCR in breast cancer patients undergoing NAC, and a PIV−based nomogram achieved excellent discrimination with an AUC of 0.815 in the validation cohort (28). Another study showed that higher PIV is significantly associated with shorter progression−free survival in breast cancer patients with lymph node metastasis receiving neoadjuvant therapy, with AUCs of 0.867, 0.802, and 0.853 for predicting 1−, 2−, and 3−year PFS, respectively (29). Previous studies have mainly focused on baseline inflammatory indices or dynamic changes such as dNLR (30) and dSII (31), whereas our results suggest that the absolute PIV value after two cycles may provide more informative insight into evolving therapeutic resistance.

Importantly, our study focused on non−response rather than pCR. Early identification of patients unlikely to achieve meaningful tumor regression is directly actionable, given that non−response (MP grades 1–3) independently predicts worse survival (32, 33). In addition, unlike many recently published radiomics or deep learning models, which often suffer from limited interpretability and poor reproducibility across different imaging protocols, our approach relied on routine MRI parameters, pathological assessment, and inexpensive blood-based biomarkers. For instance, an integrated radiomics and deep learning model achieved an AUC of 0.892 on internal testing but dropped to 0.825 on external validation (34), reflecting the generalizability challenges of these complex models. A longitudinal radiomics approach also showed predictive capability for RCB scores, yet its reliance on high-dimensional features with limited biological transparency poses barriers to clinical adoption (35). In contrast, the variables in our model are directly measurable and biologically interpretable, which may facilitate clinical accessibility and prospective validation in future studies.

Several limitations should be acknowledged. First, this was a retrospective single-center study with a relatively limited sample size, particularly regarding the number of non-response events, which may increase the risk of model overfitting and optimistic performance estimation despite internal validation procedures. Although LASSO regularization, five-fold cross-validation, bootstrap-based calibration analysis, and decision curve analysis were applied to improve model robustness, the proposed model still requires validation in larger multicenter external cohorts before potential clinical application. Second, external validation was not available in the present study. Because this multimodal framework required simultaneous availability of longitudinal MRI parameters, tumor microenvironment biomarkers (including TILs and TSR), and clinicopathological data, assembling independent external datasets with comparable imaging protocols and pathological assessments was challenging. Therefore, the current study should be considered exploratory and hypothesis-generating. Third, the cohort included biologically heterogeneous breast cancer subtypes and different neoadjuvant treatment regimens. Although treatment category distributions differed between groups, treatment selection was largely guided by molecular subtype and contemporary clinical practice. To reduce potential multicollinearity and instability caused by highly heterogeneous regimens within a limited sample size, treatment category was not incorporated into the final predictive model. Fourth, BPE grading and MRI kinetic interpretation remain partially observer dependent. We therefore performed a formal inter-observer agreement analysis, which demonstrated substantial to almost perfect reproducibility for MRI features. However, all final imaging variables were determined by consensus, which may mask pre-consensus variability. For pathological assessment, intra-observer agreement was almost perfect, but inter-observer generalizability remains to be confirmed. Future studies incorporating digital pathology, AI-based imaging analysis, and prospective reproducibility assessment may further improve methodological robustness. Finally, feature selection was performed prior to cross-validation rather than fully nested within each fold, which may have introduced potential information leakage and optimistic bias. Future prospective multicenter studies should incorporate fully nested validation frameworks and independent external validation strategies to further confirm the generalizability and clinical utility of the proposed model.

In conclusion, a multimodal prediction model integrating clinicopathological, imaging, tumor microenvironment, and systemic inflammatory features showed potential for early identification of breast cancer patients unlikely to benefit from neoadjuvant therapy. These exploratory findings require external validation in larger multicenter cohorts before clinical application.

## Data Availability

The datasets generated and analyzed during the current study are available from the corresponding author on reasonable request.

## References

[1] Loibl S André F Bachelot T Barrios CH Bergh J Burstein HJ . Early breast cancer: ESMO clinical practice guideline for diagnosis, treatment and follow-up. Ann Oncol. (2024) 35:159–82. doi: 10.1016/j.annonc.2023.11.016 38101773

[2] Spring LM Bar Y Isakoff SJ . The evolving role of neoadjuvant therapy for operable breast cancer. J Natl Compr Canc Netw. (2022) 20:723–34. doi: 10.6004/jnccn.2022.7016 35714678

[3] Yau C Osdoit M van der Noordaa M Shad S Wei J de Croze D . Residual cancer burden after neoadjuvant chemotherapy and long-term survival outcomes in breast cancer: a multicentre pooled analysis of 5161 patients. Lancet Oncol. (2022) 23:149–60. doi: 10.1016/s1470-2045(21)00589-1 34902335 PMC9455620

[4] Janssen LM den Dekker BM Gilhuijs KGA van Diest PJ van der Wall E Elias SG . MRI to assess response after neoadjuvant chemotherapy in breast cancer subtypes: a systematic review and meta-analysis. NPJ Breast Cancer. (2022) 8:107. doi: 10.1038/s41523-022-00475-1 36123365 PMC9485124

[5] Fowler AM Mankoff DA Joe BN . Imaging neoadjuvant therapy response in breast cancer. Radiology. (2017) 285:358–75. doi: 10.1148/radiol.2017170180 29045232

[6] Fiste O Mavrothalassitis E Kokkalis A Anagnostakis M Gomatou G Kontogiannis A . Inflammation-related biomarkers as predictors of pathological complete response in early-stage breast cancer. Clin Transl Oncol. (2025) 27:2453–60. doi: 10.1007/s12094-024-03814-9 39668275

[7] Ozkan EM Karadag I Inanc M Ozkan M . Dynamic immune-nutritional indices as powerful predictors of pathological complete response in patients with breast cancer undergoing neoadjuvant chemotherapy. J Clin Med. (2026) 15. doi: 10.3390/jcm15020418 41598362 PMC12842594

[8] Pislar N Gasljevic G Matos E Pilko G Zgajnar J Perhavec A . Predicting nodal response to neoadjuvant treatment in breast cancer with core biopsy biomarkers of tumor microenvironment using data mining. Breast Cancer Res Treat. (2025) 210:87–94. doi: 10.1007/s10549-024-07539-9 39496911 PMC11787214

[9] Xinsen L Yang K Bingzhi C Xiuhong C Xinling L Xinyao X . Vague-segment technique: Automatic computation of tumor stroma ratio for breast cancer on whole slides. IEEE J BioMed Health Inform. (2024) 28:905–16. doi: 10.1109/jbhi.2023.3341101 38079367

[10] Li L Yang P Hong C Chen D Lian W . Predictive value of tumor-infiltrating lymphocytes for neoadjuvant therapy response and prognosis in breast cancer: a multicenter retrospective study based on Chinese population. BMC Cancer. (2025) 25:1585. doi: 10.1186/s12885-025-15022-x 41094431 PMC12522574

[11] Karancsi Z Hagenaars SC Németh K Mesker WE Tőkés AM Kulka J . Tumour-stroma ratio (TSR) in breast cancer: comparison of scoring core biopsies versus resection specimens. Virchows Arch. (2024) 485:703–16. doi: 10.1007/s00428-023-03555-0 37198327 PMC11522047

[12] Hagenaars SC de Groot S Cohen D Dekker TJA Charehbili A Meershoek-Klein Kranenbarg E . Tumor-stroma ratio is associated with Miller-Payne score and pathological response to neoadjuvant chemotherapy in HER2-negative early breast cancer. Int J Cancer. (2021) 149:1181–8. doi: 10.1016/s0959-8049(20)30819-4 PMC8362217 34043821

[13] Cao Y Wang X Li L Shi J Zeng X Huang Y . Early prediction of pathologic complete response of breast cancer after neoadjuvant chemotherapy using longitudinal ultrafast dynamic contrast-enhanced MRI. Diagn Interv Imaging. (2023) 104:605–14. doi: 10.1016/j.diii.2023.07.003 37543490

[14] Wu Q Zhu M Xie H Geng X Wang Y Wu Z . Dynamic evolution of vascular features based on magnetic resonance imaging to predict pathological response, patterns of recurrence and survival outcomes in breast cancer neoadjuvant chemotherapy. Curr Oncol. (2025) 32. doi: 10.3390/curroncol32060350 40558293 PMC12192275

[15] Xiao J Rahbar H Hippe DS Rendi MH Parker EU Shekar N . Dynamic contrast-enhanced breast MRI features correlate with invasive breast cancer angiogenesis. NPJ Breast Cancer. (2021) 7:42. doi: 10.1038/s41523-021-00247-3 33863924 PMC8052427

[16] Ma W Yang L Zhang Y Gao Y Jie H Huang C . Heterogeneity assessment of breast cancer tumor microenvironment: multiparametric quantitative analysis with DCE-MRI and discovery of radiomics biomarkers. Breast Cancer (Dove Med Press). (2025) 17:573–81. doi: 10.2147/bctt.s530834 40657027 PMC12255257

[17] Adrada BE Guirguis MS Huo L Yam C Tripathy D Candelaria R . Imaging- and tumor biomarker-based multivariable model for early prediction of pathologic complete response to neoadjuvant systemic therapy in triple-negative breast cancer. JCO Precis Oncol. (2025) 9:e2500410. doi: 10.1200/po-25-00410 41411609 PMC12716372

[18] Thomas J Malla L Shibwabo B . Advances in analytical approaches for background parenchymal enhancement in predicting breast tumor response to neoadjuvant chemotherapy: a systematic review. PLoS One. (2025) 20:e0317240. doi: 10.1371/journal.pone.0317240 40053513 PMC11888135

[19] Cho SM Eom HJ Kim HJ Choi WJ Chae EY Shin HJ . Background parenchymal enhancement as a predictor of invasive disease-free survival in hormone receptor-positive, HER2-negative breast cancer. Eur J Radiol. (2025) 191:112340. doi: 10.1016/j.ejrad.2025.112340 40743873

[20] Ragusi MAA van der Velden BHM Meeuwis C Tetteroo E Coerkamp EG van Nijnatten TJA . Long-term survival in breast cancer patients is associated with contralateral parenchymal enhancement at MRI: outcomes of the SELECT study. Radiology. (2023) 307:e221922. doi: 10.1148/radiol.221922 36975820

[21] Ul Ain N Ishaq S Islam MK Badar Q Chaudhry K Adil T . Association of tumor-infiltrating lymphocytes (TILs) with pathological complete response to neoadjuvant therapy in breast cancer. Cureus. (2025) 17:e96076. doi: 10.7759/cureus.96076 41356925 PMC12676981

[22] Bear HD Deng X Bandyopadhyay D Idowu M Jenkins TM Kmieciak M . T-cell immune checkpoint inhibition plus hypomethylation for locally advanced HER2-negative breast cancer: a phase 2 neoadjuvant window trial of decitabine and pembrolizumab followed by standard neoadjuvant chemotherapy. J Immunother Cancer. (2025) 13. doi: 10.1136/jitc-2024-010294 40021215 PMC11873355

[23] Okcu O Öztürk >Ç Yalçın AC Şen B Yalçın N Hacıhasanoğlu E . Tumor-stroma type and tumor-stroma ratio predict neoadjuvant chemotherapy response in breast cancer. Rev Assoc Med Bras (1992). (2025) 71:e20241225. doi: 10.1590/1806-9282.20241225 40172391 PMC11964313

[24] Andour L Hagenaars SC de Groot AF Krol-Warmerdam EMM Kroep JR Hazelbag HM . The predictive value of the tumor-stroma ratio for neoadjuvant endocrine therapy in hormone receptor-positive breast cancer. Int J Cancer. (2026). doi: 10.1002/ijc.70490 41964157 PMC13284621

[25] Cha YJ Ahn SG Bae SJ Yoon CI Seo J Jung WH . Comparison of tumor-infiltrating lymphocytes of breast cancer in core needle biopsies and resected specimens: a retrospective analysis. Breast Cancer Res Treat. (2018) 171:295–302. doi: 10.1007/s10549-018-4842-7 29869774

[26] Le MK Odate T Kawai M Oishi N Kondo T . Investigating the role of core needle biopsy in evaluating tumor-stroma ratio (TSR) of invasive breast cancer: a retrospective study. Breast Cancer Res Treat. (2023) 197:113–21. doi: 10.1007/s10549-022-06768-0 36335529

[27] Li F Chen H Lu X Wei Y Zhao Y Fu J . Combining the tumor-stroma ratio with tumor-infiltrating lymphocytes improves the prediction of pathological complete response in breast cancer patients. Breast Cancer Res Treat. (2023) 202:173–83. doi: 10.1007/s10549-023-07026-7 37528265

[28] Tian Z Xi Y Chen M Hu M Chen F Wei L . Construction of a nomogram model for predicting pathologic complete response in breast cancer neoadjuvant chemotherapy based on the pan-immune inflammation value. Curr Oncol. (2025) 32. doi: 10.3390/curroncol32040194 40277751 PMC12026318

[29] Wang Z Wu X Yang L Guo Y Zeng X Yan X . Prognostic value of pan-immune-inflammation in breast cancer patients with lymph node metastasis and neoadjuvant therapy. Med (Baltimore). (2025) 104:e45713. doi: 10.1097/md.0000000000045713 41305711 PMC12643588

[30] Ocaña A Chacón JI Calvo L Antón A Mansutti M Albanell J . Derived neutrophil-to-lymphocyte ratio predicts pathological complete response to neoadjuvant chemotherapy in breast cancer. Front Oncol. (2021) 11:827625. 35223459 10.3389/fonc.2021.827625 PMC8875201

[31] Yüksel C Doğan M Çulcu S Doğan L . Can we adapt neoadjuvant rectal (NAR) score with systemic immune-inflammation index (SII) and prognostic nutritional index (PNI) as prognostic factors in locally advanced breast cancer? Breast Cancer (Dove Med Press). (2026) 18:578298. 41743195 10.2147/BCTT.S578298 PMC12932039

[32] Shao Y Guan H Luo Z Yu Y He Y Chen Q . Predictive factors for outcome in HER2-low breast cancer patients after neoadjuvant chemotherapy. Front Oncol. (2025) 15:1459444. doi: 10.3389/fonc.2025.1459444 40110191 PMC11920645

[33] Huang Z Liu Y Li S Li Y Wu Z He H . IHC4 and COMBINE scores for enhanced prognostic stratification in HR+/HER2- breast cancer patients after neoadjuvant chemotherapy. Breast Cancer Res Treat. (2025) 211:307–19. doi: 10.1007/s10549-025-07645-2 39954110 PMC12006200

[34] Zhang Y Cai J Cui C Qi S Zhao D . Predicting breast cancer response to neoadjuvant therapy by integrating radiomic and deep-learning features from early-and-peak phases of DCE-MRI. BMC Cancer. (2025) 25:1747. doi: 10.1186/s12885-025-15095-8 41219899 PMC12604257

[35] Li W Huang Y Zhu T Ye G Wang K . Multiparametric MRI-based longitudinal-radiomics analysis for early prediction of treatment response of breast cancers to neoadjuvant chemotherapy. Appl Radiat Isot. (2026) 227:112278. doi: 10.1016/j.apradiso.2025.112278 41205319

