# Supplementary material for: Early identification of neoadjuvant therapy non-response via multimodal immune-imaging biomarkers in breast cancer
Source: Front Immunol. 2026 Jun 10;17:1877547. doi: 10.3389/fimmu.2026.1877547 (PMC13290805; doi:10.3389/fimmu.2026.1877547)
Supplement: Supplementary file 1 [file DataSheet1.pdf]

Supplementary Table S1. Detailed neoadjuvant treatment regimens.

Treatment category	Specific regimens
Anthracycline–taxane sequential regimens	EC–T, AC–T, EC–wTP, wTP–EC
HER2-targeted regimens	EC–THP, AC–THP, TCbHP, TCbPH, TCbH, TPH
Platinum/taxane-based regimens	wTP, TP, wTP–HPy
Other regimens	TX, TC–NE, wTCb–EN

Note: EC, epirubicin + cyclophosphamide; AC, doxorubicin + cyclophosphamide; T, docetaxel; wTP, weekly paclitaxel + carboplatin; THP, docetaxel + trastuzumab + pertuzumab; TCbHP, docetaxel + carboplatin + trastuzumab + pertuzumab; TCbPH, docetaxel + carboplatin + pertuzumab + trastuzumab (differing sequence); TCbH, docetaxel + carboplatin + trastuzumab; TPH, docetaxel + pertuzumab + trastuzumab; HPy, trastuzumab + pertuzumab + pyrotinib; TX, docetaxel + capecitabine; TC, docetaxel + cyclophosphamide; NE, vinorelbine + epirubicin; wTCb, weekly paclitaxel + carboplatin; EN, epirubicin + vinorelbine.

Supplementary Table S2. Inter-observer and intra-observer reproducibility analysis.

Variable	Metric	Value (95% CI)	Interpretation
EER	ICC	0.74 (0.65–0.81)	Substantial
PER	ICC	0.70 (0.60–0.78)	Substantial
LER	ICC	0.72 (0.63–0.80)	Substantial
TTP	ICC	0.79 (0.71–0.85)	Substantial
ADC	ICC	0.82 (0.75–0.88)	Almost perfect
EER2	ICC	0.76 (0.67–0.83)	Substantial
PER2	ICC	0.72 (0.63–0.80)	Substantial
LER2	ICC	0.74 (0.65–0.81)	Substantial
TTP2	ICC	0.81 (0.73–0.87)	Almost perfect
ADC2	ICC	0.84 (0.77–0.89)	Almost perfect

Variable	Metric	Value (95% CI)	Interpretation
BPE	Cohen's κ	0.74 (0.63–0.84)	Substantial
Lesion type	Cohen's κ	0.86 (0.79–0.93)	Almost perfect
Peritumoral edema	Cohen's κ	0.79 (0.69–0.88)	Substantial
Intratumoral necrosis	Cohen's κ	0.81 (0.72–0.89)	Almost perfect
Post-2nd BPE change	Cohen's κ	0.76 (0.65–0.86)	Substantial
Post-2nd Edema	Cohen's κ	0.77 (0.67–0.86)	Substantial
Post-2nd Necrosis	Cohen's κ	0.80 (0.71–0.89)	Almost perfect
Baseline TIC	Weighted κ	0.79 (0.69–0.87)	Substantial
Post-2nd TIC	Weighted κ	0.80 (0.72–0.88)	Substantial
TILs	Cohen's κ	0.87 (0.80–0.94)	Almost perfect
TSR	Cohen's κ	0.83 (0.75–0.91)	Almost perfect

Note: ICC, intraclass correlation coefficient (two-way random-effects, absolute agreement, single rater); Weighted κ, weighted kappa with quadratic weights. EER, early enhancement rate; PER, peak enhancement rate; LER, late enhancement rate; TTP, time to peak; ADC, apparent diffusion coefficient; BPE, background parenchymal enhancement; TIC, time-intensity curve; TILs, tumor-infiltrating lymphocytes; TSR, tumor-stroma ratio. Variables with "2" denote measurements after two cycles of neoadjuvant therapy. MRI reproducibility was assessed by inter-observer agreement between two independent radiologists; pathological reproducibility was assessed by intra-observer agreement of the same pathologist with a 4-week washout period. Interpretation of agreement strength follows Landis and Koch (1977): 0.61–0.80 substantial, 0.81–1.00 almost perfect.

Supplementary Table S3. Variance inflation factor (VIF) analysis of the multimodal model.

Variable	VIF
HER2	1.273

Variable	VIF
ER	1.046
Ki-67	1.106
LER2	1.252
BPE	1.091
TTP2	1.140
TSR	1.304
TILs	1.273
PIV2	1.110

Note: VIF, variance inflation factor. All VIF values were below 1.5, indicating no significant multicollinearity among the variables retained in the final multimodal model. A VIF threshold of < 5 (or < 10) is conventionally accepted for excluding problematic collinearity.

Supplementary Table S4. Bootstrap variable stability analysis across 200 resamples.

Variable	Selection frequency
TILs	0.990
HER2	0.980
Ki-67	0.935
TSR	0.910
TTP2	0.855
ER	0.840
LER2	0.785
BPE	0.735
PIV2	0.710

Note: LASSO regression was performed on 200 bootstrap samples using all 12 candidate variables retained from the four individual domain models. For each variable, the selection frequency indicates the proportion of resamples in which the variable had a non-zero coefficient. Variables are listed in descending order of selection frequency. The nine variables with selection frequency ≥ 0.710 were retained in the final multimodal model. The three variables with lower selection frequencies (dSIRI, ADC, SIRI2) were excluded by LASSO on the original dataset, consistent with their lower stability across resamples.

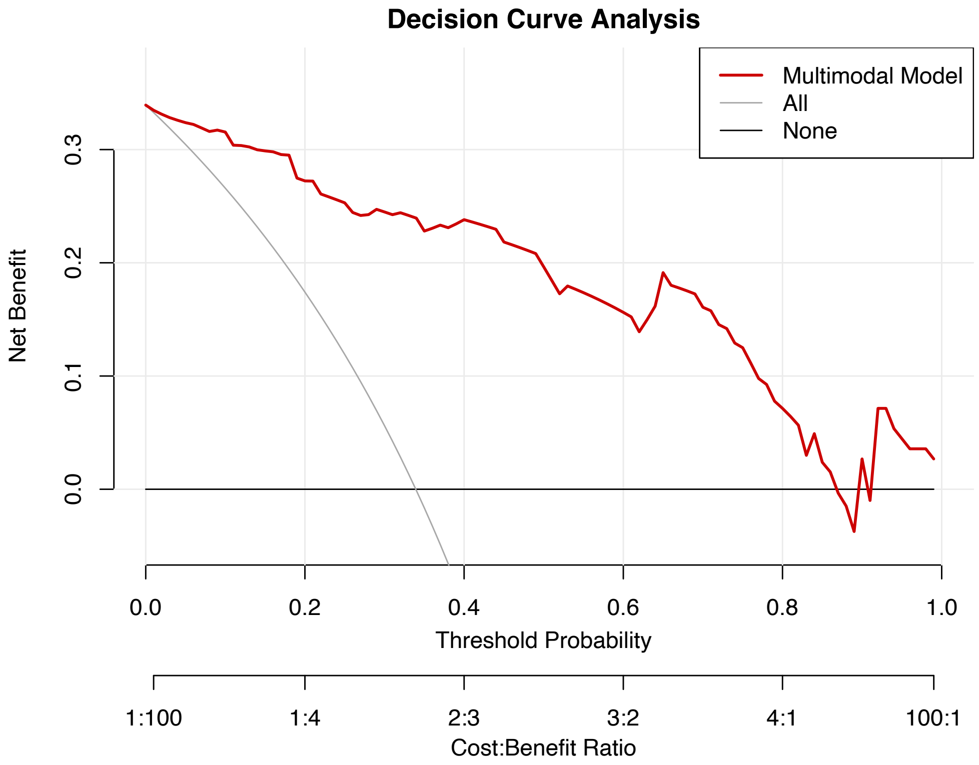

Supplementary Figure S1 Decision curve analysis of the multimodal model

The decision curve shows the net benefit of the multimodal model (red line) across a range of threshold probabilities for predicting neoadjuvant therapy non-response. The gray horizontal line at net benefit = 0 (“treat none” strategy) and the black dashed line (“treat all” strategy) are included for reference. The model demonstrated a favorable net clinical benefit over a broad range of clinically relevant threshold probabilities.
